# The correlation between motor impairments and event-related desynchronization during motor imagery in ALS patients

**DOI:** 10.1186/1471-2202-13-66

**Published:** 2012-06-15

**Authors:** Takashi Kasahara, Kentaro Terasaki, Yuki Ogawa, Junichi Ushiba, Harumichi Aramaki, Yoshihisa Masakado

**Affiliations:** 1 Department of Rehabilitation Medicine, Tokai University School of Medicine, Shimokasuya 143, Isehara, Kanagawa, 259-1193, Japan; 2 Faculty of Science and Technology, Keio University, 3-14-1 Hiyoshi, Kouhoku-ku, Yokohama, Kanagawa, 223-8522, Japan; 3 Department of Rehabilitation Medicine, National Hokone Hospital, 412 Kazamatsuri, Hokone, Kanagawa, 250-0032, Japan

**Keywords:** Brain-computer interface, Amyotrophic lateral sclerosis, Bulbar dysfunction

## Abstract

**Background:**

The event-related desynchronization (ERD) in EEG is known to appear during motor imagery, and is thought to reflect cortical processing for motor preparation. The aim of this study is to examine the modulation of ERD with motor impairment in ALS patients. ERD during hand motor imagery was obtained from 8 ALS patients with a variety of motor impairments. ERD was also obtained from age-matched 11 healthy control subjects with the same motor task. The magnitude and frequency of ERD were compared between groups for characterization of ALS specific changes.

**Results:**

The ERD of ALS patients were significantly smaller than those of control subjects. Bulbar function and ERD were negatively correlated in ALS patients. Motor function of the upper extremities did was uncorrelated with ERD.

**Conclusions:**

ALS patients with worsened bulbar scales may show smaller ERD. Motor function of the upper extremities did was uncorrelated with ERD.

## Background

Amyotrophic lateral sclerosis (ALS) is a progressive neurodegenerative disorder characterized by loss of upper and lower motor neurons. This disease may finally leave patients with no voluntary muscle control, and with minimal or no means of communication. The capacity to communicate with family members and other caregivers is a critical component in maintaining quality of life [1]. Thus, these patients need communication technologies that do not require neuromuscular function [2].

The Brain-Computer Interface (BCI) is a technology that aims to transform thought into action using the electrical activity generated by ensembles of cortical neurons [3]. This has been made possible due to advances in methods of electroencephalography (EEG) analysis and in information technology, associated with a better understanding of the functional significance of certain EEG parameters [4]. Berger found motor execution or imagery can block or desynchronize the ongoing EEG activity [5]. These event-related phenomena represent frequency specific changes in ongoing EEG activity and may consist of decreases of power in given frequency bands. This may be due to a decreasing in synchrony of the underlying neural populations, called event-related desynchronization (ERD) [6], [7]. ERD is interpreted as an electrophysiological correlate of activated cortical areas involved in the processing of sensory or cognitive information or the production of motor behavior [8]. In general, this phenomenon is recognized in sensorimotor areas of both hemispheres with motor execution or motor imagery, but the contralateral hemisphere is dominant [4], [9]. Neuper and colleagues reported this lateralization is reinforced by training with feedback [4], and they made highly accurate classifications with the BCI system using ERD.

The so-called Graz-BCI, developed by Pfurtscheller’s group at the Graz University of Technology in the early nineties, was the first online BCI system using ERD classification in single EEG trials to discriminate between different types of motor execution and motor imagery [10], [11]. Since then, several reports have used ERD to design BCI systems as communication devices for severely handicapped patients, based on motor imagery.

Many different disorders, such as stroke, muscle dystrophy, high spinal cord injury, and ALS may lead to severe motor paralysis. As previously mentioned, ALS is a rapidly progressive neurodegenerative disease of unknown etiology. In this aspect, ALS is distinct from other chronic diseases. Clinically, the disease course is progressive, ultimately resulting in death or mechanical ventilation in the vast majority of patients [12]. Since the progression of weakness is rapid and drastic, ALS patients may not have sufficient time for training with a BCI system. In order to apply the BCI system using ERD classification without elaborate training in ALS patients, it is need to analyze the correlation between progressive impairments and ERD with motor imagery.

However, there have been few reports about this relationship. Kübler and colleagues investigated the relationship between general physical impairments and BCI performance, but they did not evaluate impairments like muscle weakness, bulbar symptoms, and activities of daily living (ADL) [13]. They also did not analyze the correlation between ERD by itself and these impairments. It might be difficult to determine the practical and effective BCI system for an ALS patient without basic evaluation of how the progressive impairments due to neurodegenerative changes affect the ERD. We thought it is indispensable to analyze the specificity of EEG with ALS for realize the effective BCI design.

In this paper, we investigated features of ERD with ALS patients and healthy subjects with hand grasping motor imagery. We believed that it would be difficult for patients in advanced stages of ALS to make vivid hand grasping imagery as they would not have actually grasped for a long time. So we hypothesized that weaker strength of the upper extremities would yield smaller ERD in ALS. This paper attempts to evaluate these impairments of ALS patients using the modified Norris ALS scale [14], and then to analyze potential correlations between the ERD and these impairments. We investigated the lateralization of ERD as well as the significant frequency for characterization of EEG during motor imagery.

## Results

### Experiment 1

#### Impairments and ERD of ALS patients

Table 1 shows the modified Norris ALS scale, magnitude of ERD, and the frequencies where ERD was observed with right or left hand imagery in 8 ALS patients. The manual muscle test (MMT) of the left upper extremity (mean ± SD) was 13.6 ± 7.0 (range 6–29), and the right was 13.1 ± 6.0 (6–24). Bulbar scale was 23.1 ±11.8 (2–38), and ADL (limb scale) was 15.5 ± 14.5 (2–43). ERD and frequencies (mean ± SD) picked up from the electrodes of C3 and C4 Laplacian during the left hand imagery were 87.1 ±7.0 (74–98), 15.5 ± 4.2 (9–21) and 85.9 ± 8.9 (71–95), 15.1 ± 4.2 (8–21), respectively. Those with right hand imagery were 85.1 ± 11.0 (64–98), 18.3 ± 6.8 (12–26), and 83.3 ± 10.1 (67–95), 15.9 ± 4.0 (11–24), respectively. Almost ALS patients complained of fatigue and requested breaks at least once throughout every session.

**Table 1:** Impairment scales, ERD, and frequencies during imagery for ALS patients

**ALS**	**Lt U/E MMT**	**Rt U/E MMT**	**Bulbar scale**	**ADL (limb scale)**	**Lt hand imagery**				**Rt hand imagery**			
					**ERD at C3**	**F**	**ERD at C4**	**F**	**ERD at C3**	**F**	**ERD at C4**	**F**
1	6	6	38	14	74	18	71	14	64	12	71	15
2	10	10	33	4	87	13	84	19	74	26	82	13
3	12	24	32	32	82	20	75	21	85	12	67	19
4	29	20	21	43	87	17	86	18	90	19	90	24
5	11	11	12	6	88	9	91	13	93	26	90	16
6	10	10	25	2	89	14	90	16	87	12	91	14
7	14	14	2	10	98	12	95	12	98	13	95	11
8	17	10	22	13	92	21	95	8	90	26	80	15
mean	13.6	13.1	23.1	15.5	87.1	15.5	85.9	15.1	85.1	18.3	83.3	15.9
SD	7.0	6.0	11.8	14.5	7.0	4.2	8.9	4.2	11.0	6.8	10.1	4.0

**Abbreviations:** U/E MMT, upper extremity’s MMT; ERD at C3/4, ERD at C3/4 (%); F, frequency (Hz).

#### ERD in control group

The ERD and frequencies (mean ± SD) picked up from the electrodes around C3 or C4 with surface Laplacian derivation methods are shown in Table 2 for right and left hand imagery in 11 controls. During left hand imagery, ERD and frequencies picked up from the electrodes of C3 and C4 Laplacian were 67.4 ± 18.4 (37–96), 14.7 ± 4.5 (8–24) and 62.8 ± 17.9 (35–84), 14.9 ± 4.3 (8–24) respectively. With right hand imagery, the respective measures were 66.8 ± 20.8 (29–96), 15.9 ± 4.7 (9–24), and 69.5 ± 20.8 (27–92), 12.6 ± 4.3 (8–21).

**Table 2:** ERD and frequencies during imagery for controls

**control**	**Lt hand imagery**				**Rt hand imagery**			
	**ERD at C3**	**F**	**ERD at C4**	**F**	**ERD at C3**	**F**	**ERD at C4**	**F**
1	54	10	38	8	58	9	55	9
2	37	15	35	14	29	15	40	13
3	78	18	64	13	73	15	82	13
4	89	15	82	12	96	13	75	10
5	64	24	84	11	83	10	87	8
6	57	10	41	14	35	15	27	15
7	96	8	78	24	73	15	92	21
8	67	15	62	16	85	23	71	17
9	71	14	71	16	63	16	70	8
10	45	18	59	17	59	20	77	16
11	83	15	77	19	81	24	89	9
mean	67.4	14.7	62.8	14.9	66.8	15.9	69.5	12.6
SD	18.4	4.5	17.9	4.3	20.8	4.7	20.8	4.3

**Abbreviations:** ERD at C3/4, ERD at C3/4 (%); F, frequency (Hz).

#### Comparison of ERD and frequencies between hemispheres within group and between ALS and control groups

In ALS patients, there was no significant difference in averaged ERD or frequencies between C3 Laplacian and C4 Laplacian during either left or right hand imagery, as summarized in Table 3. Similarly, there were no significant differences across hemispheres in these measures for the control subjects.

**Table 3:** Comparison of ERD and its frequencies across hemispheres within each group and between the ALS and control groups

**A. ALS (n= 8)**	**Lt hand imagery**		**p**	**Rt hand imagery**		**p**
	**C3**	**C4**		**C3**	**C4**	
ERD (%)	87.1±7.0	85.9± 8.9	0.35	85.1±11.0	83.3±10.1	0.74
Frequency (Hz)	15.5± 4.2	15.1±4.2	0.67	18.3± 6.8	15.9± 4.0	0.53
control	Lt hand imagery		p	Rt hand imagery		p
(n= 11)	C3	C4		C3	C4	
ERD (%)	67.4±18.4	62.8±17.9	0.22	66.8±20.8	69.5±20.8	0.45
Frequency (Hz)	14.7± 4.5	14.9±4.3	0.96	15.9± 4.8	12.6± 4.3	0.06
(Wilcoxon sign rank test)						
**B.** group		ALS (n= 8)		Control (n= 11)		p
Age (years)		66.3 ±13.5		67.0 ± 3.7		0.84
ERD (%)		85.3±9.0		66.6±19.0		0.00
Frequency (Hz)		16.2±4.9		14.5±4.5		0.23
Significant results are highlighted in bold				Degree of freedom: 19 (Mann-Whitney *U* test)		

**A:** ERD and its frequencies in each hemisphere within each group.

**B:** Age, ERD, and its frequencies compared between ALS and control groups.

We compared mean age, mean magnitude of ERD, and frequencies between ALS and control groups. The ERD of ALS patients were significantly smaller than those of controls (*u* = 259, p < 0.01). There was no difference between the two groups in age and or in the frequencies that showed ERD (Table 3).

#### Possible correlation between ERD and frequency

We analyzed the frequency that showed ERD in all subjects. In this study, we defined the alpha band as the frequency range from 8 to 13 Hz and the beta band as the range from 14 to 30 Hz.

**Figure 1:**
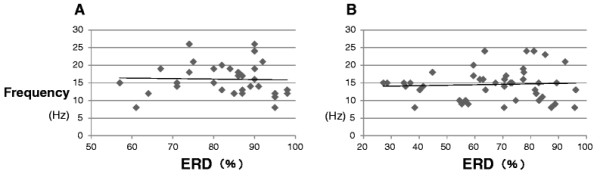
**Correlation between ERD magnitude and the frequency showing ERD. A: ALS patients**. **B: controls.** There were no correlations between ERD and frequency in either group.

In both groups, about 60% showed ERD in the beta frequency band. There were no correlations between ERD and frequency in either the ALS patients or controls (Figure 1A and B).

#### Correlation between characteristics, impairments, and ERD in ALS patients

**Figure 2:**
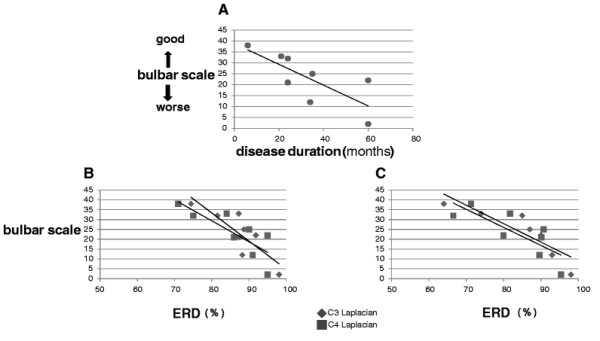
**Correlation between bulbar scale and ERD of ALS patients. A**: **Correlation between bulbar scale and disease duration**. **B, C.** Correlation between bulbar scale and ERD of ALS patients with left and right hand imagery. B. **Left hand imagery.** C. **Right hand imagery**. The more worsen bulbar scale, the smaller ERD.

We analyzed the correlations between ERD, the subjects’ characteristics, and the modified Norris scale in ALS patients. As clinically known, disease duration and bulbar scale were negatively correlated (Figure 2A). Longer disease durations correlated with worse bulbar scale scores. The bulbar scale scores and ERD were negatively correlated both in C3 and C4 Laplacian locations during left and right hand imagery, respectively (Figure 2B and C). A worse bulbar scale score predicted a smaller ERD. On the other hand, there were no correlations between ERD and age, upper extremities MMT, or ADL. Without C4 Laplacian ERD during right hand imagery, disease duration and ERD were negatively correlated (Table 4).

**Table 4:** Correlations between ERD and the characteristics and impairments of ALS patients

		**Lt hand imagery**		**Rt hand imagery**	
		**ERD at C3**	**ERD at C4**	**ERD at C3**	**ERD at C4**
Age	ρ	0.28	0.25	0.26	0.40
	p value	0.51	0.55	0.53	0.33
Disease duration	ρ	0.94	0.93	0.80	0.55
	p value	**0.00**	**0.00**	**0.02**	0.16
Lt U/E MMT	ρ	0.43	0.54	0.66	0.18
	p value	0.29	0.17	0.07	0.67
Rt U/E MMT	ρ	0.05	0.11	0.47	0.10
	p value	0.91	0.79	0.24	0.82
Bulbar scale	ρ	-0.74	-0.83	-0.99	-0.71
	p value	**0.03**	**0.01**	**0.00**	**0.05**
ADL (limb scale)	ρ	-0.46	-0.32	-0.06	-0.49
	p value	0.26	0.43	0.89	0.22
Significant results are highlighted in bold (Spearman correlation coefficient)					

**Abbreviations:** U/E MMT, upper extremity MMT.

### Experiment 2

#### Longitudinal analysis of impairments and ERD of 2 ALS patients and 2 controls

Longitudinal measurements of the modified Norris scale, ERD, and frequencies with right or left hand imagery are shown in Table 5 and 6 for 2 ALS patients and 2 controls.

**Table 5:** Longitudinal analysis of impairments scale and ERD of 2 ALS patients

**ALS**	**M**	**Lt U/E MMT**	**Rt U/E MMT**	**Bulbar scale**	**ADL (limb scale)**	**Lt hand imagery**				**Rt hand imagery**			
						**ERD at C3**	**F**	**ERD at C4**	**F**	**ERD at C3**	**F**	**ERD at C4**	**F**
1	30	11	13	20	17	80	19	57	15	86	17	61	8
	31	11	11	18	17	93	12	75	13	89	26	80	12
	34	11	11	12	6	88	9	91	13	93	26	90	16
mean	31.7	11	11.7	16.7	13.3	87	13.3	74.3	13.7	89.3	23	77.0	12
SD	2.1	0.0	1.2	4.2	6.4	6.6	5.1	17.0	1.2	3.5	5.2	14.7	4.0
2	6	6	6	38	14	76	18	72	14	64	12	71	15
	11	4	6	38	6	58	9	60	8	58	8	58	9
	14	4	4	27	3	77	10	75	9	66	8	82	8
mean	10.3	4.7	5.3	34.3	7.7	70.3	12.3	69	10.3	62.7	9.3	70.3	10.7
SD	4.0	1.2	1.2	6.4	5.7	10.7	4.9	7.9	3.2	4.2	2.3	12.0	3.8

Impairments scale and ERD of 2 ALS patients.

**Abbreviations:** M, disease duration (month); U/E MMT, upper extremities MMT; ERD at C3/4, ERD at C3/4 (%); F, frequency (Hz).

**Table 6:** ERD of 2 controls

**control**	**M**	**Lt hand imagery**				**Rt hand imagery**			
		**ERD at C3**	**F**	**ERD at C4**	**F**	**ERD at C3**	**F**	**ERD at C4**	**F**
1	0	37	15	35	14	29	15	40	13
	+7	36	15	35	14	30	15	29	14
mean		36.5	15	35	14	29.5	15	34.5	13.5
SD		0.7	0.0	0.0	0.0	0.7	0.0	7.8	0.7
2	0	54	10	38	8	58	9	55	8
	+7	58	21	48	8	72	8	65	17
mean		56	15.5	43	8	65	8.5	60	12.5
SD		2.8	7.8	7.1	0.0	9.9	0.7	7.1	6.4

**Abbreviations:** ERD at C3/4, ERD at C3/4 (%); F, frequency (Hz).

**Figure 3:**
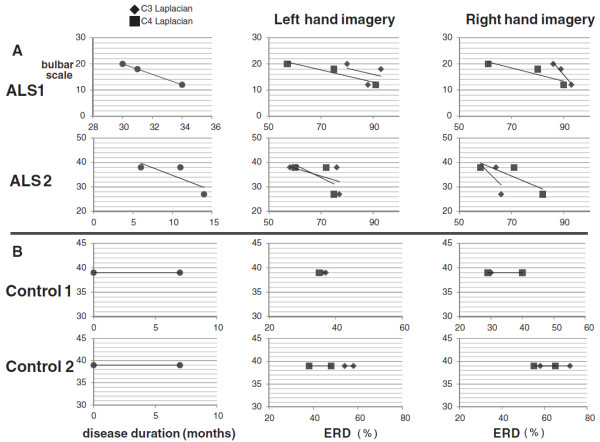
**Longitudinal analysis of correlation between bulbar scale and ERD in individuals. A:** 2 ALS patients. **B:** 2 controls. In ALS patients, disease duration and bulbar scale were negatively correlated. Bulbar scale and ERD were also negatively correlated. For controls, reproducibility of ERD was high and absolute ERD were larger than those of ALS patients.

As seen in Experiment 1, disease duration and bulbar scale were negatively correlated in ALS patients. Bulbar scale and ERD were also negatively correlated with left and right hand imagery (Figure 3A). In controls, individual ERDs were changed, but the ranges of change in ERD magnitude were small compared to ALS. Absolute ERD were larger in controls than in ALS patients (Figure 3B).

## Discussion

### Frequency of ERD as observed in ALS patients

According to the results of our research, the mean magnitude of ERD with ALS patients was smaller than that of control and about 60% of both groups showed ERD in the beta frequency band. We presumed that there would be a relationship between the magnitude of ERD and frequency. However, there were no correlations between ERD and frequency in either the ALS patients or the control subjects.

In previous reports about BCI research, alpha band components were selected as the relevant frequency with hand imagery in healthy control [2]. However, in several studies of BCI in ALS patients, beta band components were selected as the relevant frequency band [15], [16], [17], [18]. In these reports, the frequency band was selected for the sake of better classification accuracy with the BCI performance; specificity of the frequency band was not discussed at length.

We supposed that aging would affect the selection of the relevant frequency band because there was no difference between the ALS and controls on the age and frequencies that showed ERD. A progressive loss of corticospinal motor neurons during aging has been reported previously [19]. In an analysis of EEG in aging, Hamada reported that alpha band frequency (mu rhythm) decreased with age [20]. Less consistent findings have been found concerning beta desynchronization; one report indicated an increase with aging [21] while another found beta desynchronization to be unaffected [22].

We presumed that cortical degenerative changes from ALS would affect the selection of the relevant frequency band in the patients. There were few previous reports about the EEG itself with resting state in ALS patients. Mai and colleagues evaluated quantitative EEG (QEEG) in ALS patients to see if the confined cortical degeneration found in anatomical and functional examinations of central (rolandic) regions could give rise to abnormalities of cortical electrical activity [23]. QEEG in ALS patients showed a significant and well-localized decrease of alpha activity only in the central regions. They suggested that this EEG change was probably due to loss of cells in the sensorimotor cortex. We could not deny the possibility that the ERD in alpha frequency band was decreased due to neuronal loss from the progression of ALS.

The magnitude of ERD might not depend on the number of surviving cortico-motoneural cells, but rather on the concentration for the task to drive the surviving cortico-motoneural cells.

### Correlation between impairments and ERD in the ALS patients

In accord with clinical knowledge, the results of Experiment 1 showed that disease duration and bulbar scale were negatively correlated. Similarly, a worse bulbar scale indicated a smaller ERD (Figure 2A, B, and C). However, contrary to our hypothesis, the ERD was not correlated with strength of the upper extremities.

To evaluate these relationships in individual ALS patients longitudinally, Experiment 2 was conducted using the same task as Experiment 1, with measurements at three time-points. In this experiment, disease duration and ERD were both negatively correlated longitudinally with bulbar scale in ALS patients (Figure 3A). However, these tendencies were not found in controls, and the ranges of change in ERD magnitude were small (Figure 3B).

According to our results, grasping motor imagery yielding large ERD might be associated not with intact hand function or ADL, but rather with intact bulbar function.

Motor imagery is the ability to create a vivid mental image of movement [24]. A previous study reported a greater enhancement of ERD in subjects with vivid imagery than in those with non-vivid imagery [25]. Vivid imagery needs alertness and concentration. Subjects with motor deficits may have greater difficulty compared to normal subjects focusing attention on motor tasks including imagining movement [17]. ALS patients with severe bulbar dysfunction might also find it difficult to concentrate on motor imagery owing to inattention and general fatigue. Generalized tiredness is commonly a result of sleep difficulties that can arise from saliva aspiration due to severe bulbar dysfunction and positioning needs [12]. In fact, in this study and other previous reports, ALS patients complained of fatigue and requested breaks several times during the experiment [17]. We believed it was one of the reasons why the patients with worsened bulbar scale had smaller ERDs. These results suggest it is necessary to take care of drooling or tiredness of ALS patients in this state in order to use a BCI system effectively.

Several studies have shown that the act of mastication, even without calorie intake, has beneficial psychological effects, alters the state of arousal, and facilitates high scores on working memory tests [26],[27]. Sakamoto and colleagues reported mastication influenced cognitive processing time as reflected by reaction time and the latency of event-related potentials [28]. We speculated there was a possibility that a prolonged state without oral food intake due to severe dysphagia, especially in subject No 7, affected cognition and thus motor imagery indirectly.

According to the previous reports, the presence of pseudobulbar palsy in ALS is associated with frontal lobe impairment [29]. In particular, the patients with pronounced bulbar deficits could have had more breathing difficulties leading to oxygen deficit and additional impact on cognitive function [30]. However in our cases, monitoring of oxyhemoglobin saturation and evaluation of arterial blood gas was within normal, they did not use intermittent positive pressure ventilation and artificial ventilation. And patients were alert and cooperative with experiments, they were clinically diagnosed as non-demented. On the other hand, we could not evaluate their cognition with psychological battery due to severe bulbar and limb dysfunction, we could not strictly deny the possibility with presence of cognitive dysfunction that affected the ERD. Future research should be performed to confirm these relationships.

In order to find an effective BCI system using ERD classification in ALS patients, it is necessary to modify the EEG analysis system individually with the progression of symptoms for each patient. The environmental setting is indispensable for minimizing fatigue and for concentrating on imagery, especially given the progressive state of ALS patients with bulbar dysfunction.

#### Magnitude of ERD, its frequencies, and its laterality

In a previous report, right-handed subjects showed larger lateralization of ERD with right-finger movements compared to left-finger movements [31], and the right-hand movement with electrodes close to position C3 (contralateral to the imaged side) provided the highest classification accuracy for BCI control [4]. However, in our study there was no significant difference with averaged ERD between C3 Laplacian and C4 Laplacian during either left or right hand imagery in ALS patients and controls. We suggest two possible reasons for this result.

First, feedback training might be insufficient in our task. As mentioned previously, the lateralization of ERD during imagery is reinforced by training with feedback [4]. As our experiment did not involve feedback training, potential lateralization could not be reinforced.

Second, age might affect the results. In our study, mean ages of the two groups were over sixty-five years old. Using ERD analysis of the mu and beta rhythm, increased and earlier activation of the ipsilateral sensorimotor areas in older subjects has been reported [32], [33]. Labyt and colleagues reported that increased and wider spread cortical activity may be necessary to generate the correct motor program in elderly subjects compared to young subjects. They hypothesized that to compensate for a loss of specificity of subcortical inputs from the thalamus and basal ganglia, an increase of activity occurred in these subcortical nuclei. Given the above, handedness did not affect the lateralization of ERD significantly for the elderly subjects, even ALS patients, on single EEG trials without feedback training.

## Study limitations

In our study, we could not evaluate the cognition in advanced stages of ALS with psychological battery due to severe bulbar and limb dysfunction. However, it is necessary to clarify the cognition with ALS, even if in severe condition, for realize the effective BCI design. Thus, the analysis of correlation between cognitive function with pronounced bulbar deficits and the ERD magnitude awaits future studies.

## Conclusions

ALS patients with worsened bulbar scales may show smaller ERD owing to the inattention and fatigue resulting from bulbar dysfunction. Motor function of the upper extremities did was uncorrelated with ERD. The environmental setting is indispensable for minimizing fatigue and for concentrating on imagery, especially given the progressive state of ALS patients with bulbar dysfunction.

## Methods

Two experiments were conducted to investigate features of ERD during motor imagery of grasping movements. Experiment 1 was conducted to measure the features of ERD in 8 ALS patients and 11 healthy subjects with motor imagery, and to compare the ERD frequencies between ALS and control groups. To analyze the lateralization of ERD during imagery, ERD and frequencies between C3 Laplacian and C4 Laplacian were compared within both groups. Experiment 2 was conducted to investigate the reproducibility of ERD in 2 ALS patients and 2 healthy subjects longitudinally for the same task as Experiment 1. Experiment 2 was conducted with 3 measurements collected at 1- to 5-month intervals. The 4 subjects in Experiment 2 were randomly selected from the subjects in Experiment 1. A total of 19 subjects were participated in the study after giving written informed consent. The research was approved by the Clinical Research Review Committee of the School of Medicine, Tokai University, Tsurumaki Onsen Hospital, National Hokone Hospital and Faculty of Science and Technology, Keio University, Kanagawa, Japan.

### Experiment 1

The ALS group consisted of 8 consecutive unselected right-handed and non-demented patients (4 woman and 4 men) with a mean age of 66.3 ±13.5 years (range 47–84 years). Mean disease duration was 32.5 ±18.9 months (range 6–60 months). All patients were right handed. One subject (No. 7) had a tracheostomy. All patients but subject No. 7 took their main nutrition orally.

The level of clinical disability was assessed with the modified Norris ALS scale. This scale consists of 3 parts: a MMT, a bulbar scale, and a limb scale. In this study, we evaluated only the bilateral upper extremities strength with the MMT, because we used hand grasping motor imagery. The bulbar scale has 13 items to evaluate bulbar function, and the limb scale contains 21 items to evaluate extremity function that describe the capability for ADL. Full marks for each of the 3 parts were 30, 39, and 63, respectively.

Only patients fulfilling criteria for typical, sporadic (non-familial) amyotrophic lateral sclerosis were selected. All of them were diagnosed following the El Escorial criteria of the World Federation of Neurology (1994) [34]; other diseases were ruled out by clinical and instrumental examinations, according to current clinical standards. No patient had a history of cerebrovascular disease and psychiatric illness. No patient used artificial ventilation. Even though the Mini Mental State Examination (MMSE) could not be completed in these patients due to severe bulbar and limb dysfunction, they were clinically diagnosed as non-demented.

The control group consisted of 11 subjects (4 men, 7 women) with a mean age of 67 ± 3.7 years (range 61–72). All were right-handed and taking no medication. They had no medical, neurological or psychiatric illness and the results of neurological examinations were normal.

### Experimental procedure

Subjects sat in a reclining armchair with their eyes open facing a computer monitor placed approximately 80 cm in front of them at eye level. EEGs were recorded during right or left hand grasping motor imagery.

A trial started with an 8 s period of relaxation while a character indicating ‘Rest’ was presented on the monitor. After that, a character for ‘Ready’ was presented for 2 s. Then a ‘Start’ cue was displayed on the monitor and subjects were requested to imagine clenching their fist with first-person perspective for 5 s. The subjects were instructed in the task before each session [35]. The trial ended when the character for ‘Rest’ reappeared, and the next trial began after an 8 s break. One session consisted of 20 trials, and two sessions were performed in the same day with right and left hand motor imagery, respectively.

### Experiment 2

The ALS group consisted of 2 randomly selected right-handed and non-demented patients (2 men) with a mean age of 61 years (range 51–71 years). The control group consisted of 2 healthy subjects (2 women) with a mean age of 65 years (range 63–67).

### Experimental procedure

Experimental procedure and task in Experiment 2 was same as Experiment 1. However, in Experiment 2, EEG during hand grasping motor imagery was recorded and the level of clinical disability was evaluated 3 times over intervals from 1 to 5 months. Owing to the clinical status of the patients, we could not have same interval between experiments with each subject. As for ALS 1, longitudinal measurements were evaluated on the month of 30^th^, 31^th^ and 34^th^ from the onset respectively. Those with ALS 2 were evaluated on the months of 6^th^, 11^th^ and 14^th^. We longitudinally investigated the reproducibility of ERD and the correlation between the ERD and observed impairments. We also investigated the reproducibility of the ERD with a 7-month interval in 2 healthy subjects.

### Data recording

Data recording was the same procedure for both experiments. EEG recordings were made from the scalp near the sensorimotor cortex using 10 Ag/AgCI surface electrodes with a diameter of 5 mm, placed at C3 and C4 (defined by the international 10–20 system) and 20 mm frontal, back, left lateral, and right lateral positions respectively. The reference electrode was placed at right or left earlobe. An additional electrode was placed at forehead as a ground electrode. EEG signals were derived using the Hjorth transformation [36]. EMG (Electromyography) from the both right and left first dorsal interosseous (FDI) were recorded from two pairs of surface Ag/AgCl disc electrodes placed in a tendon-belly montage to confirm there was no activity during the imagery task. Impedance of the EEG and EMG electrodes was kept below 5 kΩ and 20 kΩ, respectively, during recording. The signals were amplified (g.USBamp, g.tec medical engineering GmbH), digitized (256 Hz sampling frequency) and band-pass filtered (EEG 2–70 Hz, EMG 5–100 Hz).

### Quantification of ERD

Off-line analysis of EEG data was performed using MATLAB (The Mathworks Inc.). All EEGs were used to analyze the focal activity of the cortex with surface Laplacian derivation methods. In this method, the EEG signal from each electrode was referenced to the averaged potentials from four orthogonal nearby electrodes [36].

Trials of 10 s length, spanning a time window from −5 s to +5 s with reference to the onset of motor imagery, were selected for off-line data processing. All trials were visually assessed, and trials with artifacts (resulting from eye movement or swallowing) as well as trials with EMG activity of the FDI were excluded. All trials are segmented into successive 1 s windows with 224 samples (87.5%) overlapped and the Fast Fourier transformation with a Hamming window was applied in each segment. The power spectrum density (PSD) of the each segment was estimated over the trials by Welch’s averaged periodogram method [37]^.^

ERD was defined as the decrease of PSD in relation to a 3 s reference interval before the direction of “Ready”, respectively. ERD was calculated for each time (resolution of 0.125 s) and frequency (resolution of 1 Hz) according to the following equation [9]:

(1) $$ERD(t,f)(\%)=\frac{A(t,f)}{R(f)}\times 100$$

where *A* is the PSD of the EEG at time *t* with reference to the onset of motor imagery and frequency *f* (0 s to 3 s during motor imagery), and *R* is the PSD of the baseline period (−5 s to −2 s before the starting timing of motor imagery).

The time-frequency maps of EEG with surface Laplacian derivation methods were calculated for the left hemisphere (C3 Laplacian) and the right hemisphere (C4 Laplacian). The ERD was defined as the largest power decrease during motor imagery. The ERD was calculated with bandwidths of 5 Hz each, from 8 to 30 Hz, and we defined the 5 Hz bandwidth that showed the largest ERD as the subject-specific frequency bandwidth. We defined the smallest frequency within this bandwidth of 5 Hz as the subject-specific frequency for this task.

#### Statistical analysis

All statistical analyses were carried out using statistical software (SPSS 13.0 for Windows, MapInfo Corporation, Troy, NY). Differences in ERD and frequencies between C3 Laplacian and C4 Laplacian within both groups were analyzed with Wilcoxon signed rank tests. We selected this method since we intended to analyze the within-subject factor. Differences in mean age, ERD, and frequencies in which the largest ERD showed were compared between ALS patients and controls using nonparametric Mann–Whitney U tests to analyze the between- subjects factor. The relationship between ERD and the subjects’ characteristics, impairments and between longitudinal measurements of the ERD and impairments was evaluated with the Spearman correlation coefficient.

## Abbreviations

ALS, Amyotrophic lateral sclerosis; BCI, Brain-computer interface; ERD, Event-related desynchronization; EEG, Electroencephalography; ADL, Activities of daily living; MMT, Manual muscle test.

## Competing interests

The authors declare that they have no competing interests.

## Author’s contributions

TK conceived of the experiment and was the primary investigator involved in the data collection and analysis as well as drafting of the manuscript. KT, YO, JU, and YM (senior author) contributed to the experimental design, data analysis and manuscript editing. HA contributed to the data collection. All authors read and approved the final manuscript.
